# The interplay of FLT3 and CXCR4 in acute myeloid leukemia: an ongoing debate

**DOI:** 10.3389/fonc.2023.1258679

**Published:** 2023-10-02

**Authors:** Laura Klement, Julia Drube

**Affiliations:** Institut für Molekulare Zellbiologie, CMB - Center for Molecular Biomedicine, Universitätsklinikum Jena, Friedrich-Schiller-Universität Jena, Jena, Germany

**Keywords:** acute myeloid leukemia, cancer, FLT3-ITD, stem cell niche, treatment options, CXCR4, SDF-1, CXCL12

## Abstract

FLT3 mutations are very frequent in AML and utilization of FLT3 inhibitors as approved treatment options are very common. Despite the initial success of inhibitor treatment, the development of resistances against this treatment is a major challenge in AML therapy. One of the mechanisms causing resistance is the homing of the leukemic cells in the protective niche of the bone marrow microenvironment (BMM). A pathway mediating homing to the BMM and leukemic cell survival is the CXCL12/CXCR4 axis. The analysis of patient samples in several independent studies indicated that FLT3-ITD expression led to higher CXCR4 surface expression. However, several *in vitro* studies reported contradictory findings, suggesting that FLT3-ITD signaling negatively influenced CXCR4 expression. In this commentary, we provide an overview summarizing the studies dealing with the relationship of FLT3 and CXCR4. Taken together, the current research status is not sufficient to answer the question whether FLT3 and CXCR4 act together or independently in leukemia progression. Systematic analyses in model cell systems are needed to understand the interplay between FLT3 and CXCR4, since this knowledge could lead to the development of more effective treatment strategies for AML patients.

## 1 The role of FLT3 in AML

Acute myeloid leukemia (AML) is a heterogeneous and malignant clonal disorder of the hematopoietic system. It is characterized by the uncontrolled proliferation of abnormal myeloid progenitors which are impaired in their differentiation into mature blood cells (1). Despite significant advances in therapy, AML remains a challenging disease to treat, with high relapse rates and poor overall survival.

The FMS-like tyrosine kinase 3 (FLT3, CD135) is expressed on the surface of hematopoietic stem and progenitor cells and is involved in the regulation of cell survival, proliferation, and differentiation (2–4). FLT3 plays a crucial role in the development and progression of AML as the FLT3 gene is mutated in approximately 25 to 35% of AML patients (5). The mutations are classified into two major types: internal tandem duplications (ITD) of varying length mostly in the juxtamembrane domain and point mutations of e.g., D385 or I836 in the tyrosine kinase domain (TKD) (4–6). Both types of mutations lead to ligand independent constitutive activation of the receptor, but they differ in their signal transduction: in contrast to TKD and wild type (WT) FLT3, FLT3-ITD strongly activates Signal transducer and activator of transcription 5 (STAT5) (7–9).

The ITD mutations are the most common mutations of FLT3 found in AML patients and are associated with a poor prognosis, increased relapse rates, and decreased overall survival (10, 11). The prognostic relevance of FLT3-TKD is not verified yet (6). Targeting FLT3 signaling with specific inhibitors in patients with a FLT3 mutation is a commonly used treatment strategy in AML patients (6, 12, 13). Several FLT3 inhibitors have been developed and tested in clinical trials for the treatment of AML, including quizartinib (AC220), sorafenib, or the FDA-approved drugs gilteritinib and midostaurin (PKC-412). However, despite their initial success in inducing remission and improving outcomes, the development of resistance to FLT3 inhibitors remains a major challenge (5, 12, 13).

## 2 CXCR4 as a relevant target for BMM modulation

One of the mechanisms causing resistance to inhibitor treatment is the association of leukemic cells with the bone marrow microenvironment (BMM). The bone marrow provides a protective niche for AML cells impeding the effective elimination by FLT3 inhibitors.

The stromal cells of the bone marrow produce and secrete CXCL12 [previously known as stromal cell-derived factor-1 (SDF-1)], the ligand for the C-X-C chemokine receptor type 4 (CXCR4, SDF-1 receptor, CD184). The G protein-coupled receptor CXCR4 is expressed on the surface of hematopoietic cells, where it plays a crucial role in chemotactic migration to the bone marrow, as well as cellular proliferation and survival (14). Moreover, dysregulation of CXCR4 signaling has been implicated in the pathogenesis and progression of hematopoietic malignancies and CXCR4 is widely discussed as a promising therapeutic target (14–21). The surface expression of CXCR4 was found to be of prognostic relevance in leukemia, as either low expression of CXCR4 was found to be favorable for the treatment outcome, or high expression was associated with a poor prognosis (22–26). CXCR4-positive cells can migrate to and reside in the BMM and are shielded from chemotherapeutic agents. The leukemic cells can bypass the inhibitor therapy by survival signals coming from the CXCL12/CXCR4 axis and may contribute to the development of recurrences (20, 21, 27–29). Consequently, targeting CXCR4 signaling via e.g., CXCR4 antagonists, is a potent strategy for disrupting the influence of the protective microenvironment thus enhancing the efficacy of chemotherapy in AML (29–31).

## 3 Combinatorial targeting of FLT3 and CXCR4 in AML

Since CXCR4 contributes to the survival of leukemic cells in the BMM, the combinatorial treatment of CXCR4 antagonists and FLT3 inhibitors may lead to improved therapeutic outcomes in AML. For example, Kim et al. have demonstrated that the combination of the CXCR4 antagonist LY2510924 and the FLT3 inhibitor quizartinib reversed the stroma-mediated resistance against the FLT3 inhibitor and induced the mobilization and differentiation of AML cells in mice, resulting in enhanced anti-leukemia effects (32). In addition, Borthakur et al. conducted a phase I clinical trial utilizing granulocyte colony stimulating factor (G-CSF which regulates the expression of CXCL12 in the bone marrow), the CXCR4 inhibitor plerixafor and the FLT3 inhibitor sorafenib in relapsed/refractory FLT3-ITD AML patients (NCT00943943). The combinatorial treatment provoked a mobilization of AML cells from the bone marrow to the peripheral circulation and exhibited encouraging response rates in relapsed AML patients (33). In the same line, an RNA oligonucleotide that inhibits CXCL12, showed synergistic activity in combinatorial treatment with midostaurin against FLT3-ITD-dependent leukemia cells (34). We summarized these and additional studies targeting FLT3 and CXCL12/CXCR4 simultaneously in **Table 1**.

**Table 1:** Summary of studies combining FLT3 and CXCL12/CXCR4 treatments.

Treatment	System	Publication
**GMI-1359 + sorafenib or quizartinib**	Patient-derived xenograft (PDX) mouse model with primary FLT3-ITD-AML blasts	(35)
**LY2510924 + quizartinib**	Human FLT3-ITD-AML cell line MOLM-14 Cell line-derived xenograft (CDX) mouse model with MOLM-14 cells	(32)
**Plerixafor + sorafenib + G-CSF**	Relapsed/refractory AML patients, FLT3-ITD, ≥18 years Phase I clinical trial (NCT00943943)	(33)
**CXCL12-inhibiting RNA oligonucleotide + midostaurin**	Human FLT3-ITD-AML cell lines MOLM-13 and MOLM-14	(34)
**BL-8040 + quizartinib**	Human FLT3-WT-AML cell line HL-60; human FLT3-ITD-AML cell line MV4-11; human primary AML-FLT3-ITD or -WT cells CDX mouse model with MV4-11 cells	(36)
**AMD3465 + sorafenib** **AMD3465 +/- G-CSF + sorafenib**	Murine pro-B lymphocyte line Ba/F3-ITD or Ba/F3-FLT3; human FLT3-ITD-AML cell line MOLM-13; human primary AML-FLT3-ITD cells PDX mouse model with primary FLT3-ITD-AML blasts; CDX mouse model with Ba/F3-ITD cells	(37)

A different approach, using a CXCL12 knock out mouse model also resulted in enhanced targeting of AML cells by FLT3 inhibitors and chemotherapy treatment as compared to mice with normal CXCL12 expression (38). This study describes the mitogen-activated protein kinase p38 as a possible key player in this regulation. Taken together, although targeting both FLT3 and CXCR4 simultaneously has shown promising results in several studies, the specific mechanism underlying their interplay and potential crosstalk is not yet fully understood.

## 4 Contradictory findings describing the relationship between FLT3 and CXCR4

Until now, several studies have examined the interplay of FLT3 and CXCR4. For instance, Rombouts et al. found that AML patient samples with FLT3-ITD mutation have a higher expression of surface CXCR4 (39). Similar findings were reported in a study based on the analysis of 122 patient samples in which significantly higher CXCR4 surface expression was found on cells from patients expressing FLT3-ITD than in samples expressing FLT3-WT (26). In addition, Cao et al. analyzed 466 samples from newly diagnosed AML patients as well as from healthy donors and found that cells of the healthy control group had the least, AML patients expressing FLT3-WT had medium, and AML patients with FLT3-ITD expression showed the highest CXCR4 expression (40).

In addition to the analysis of patient samples, multiple *in vitro* experiments have been conducted, but they reveal contrasting findings. Moreover, the used cell models and experimental setups are very diverse. To elucidate the current research status, the findings of several publications will be recited in the following.

In 2005, Fukuda et al. presented data where the effects of CXCL12 and FLT3 ligand (FL) co-stimulation were analyzed (41). They report that FL and CXCL12 act synergistically to mediate short term migration, and in FLT3-ITD expressing cells, the migration towards CXCL12 is strongly enhanced. Most interestingly though, long-term stimulation of FLT3-WT receptor with FL leads to a downregulation of CXCR4. Moreover, CXCR4 was downregulated in Ba/F3 or 32D cells if expressing FLT3-ITD as compared to FLT3-WT expressing cells, indicating a negative influence of FLT3 signaling on the CXCR4 surface expression (41).

In 2009, the PIM1 kinase was introduced as a possible factor influencing the CXCR4 surface expression, but not the mRNA level of CXCR4 (42). PIM1 is one of the first target genes of STAT5, which is abnormally activated in cells expressing FLT3-ITD. This leads to the assumption that FLT3-ITD could increase CXCR4 surface expression via induction of PIM1 expression. However, this study does not comment on the possible influence of FLT3-WT on the CXCR4 expression.

Shorty after, Jacobi et al. present their results: They found a reduction of CXCR4 protein and mRNA levels if FLT3-ITD was overexpressed (43). Interestingly, a co-culture of FLT3-ITD expressing cells with stromal cells led to an increased CXCR4 surface expression, higher cell growth rate, and an increase in migration. Taking this into account, the high expression of CXCR4 might lead to an overgrowth of these cells compared to cells with lower CXCR4 expression.

Cao et al. addressed the question of interplay by analyzing the CXCR4 levels in two different hematopoietic cell lines representing FLT3-ITD expression (MV4-11 cells) or FLT3-WT expression (HL-60, an acute promyelocytic cell line). In contrast to the above-mentioned studies, they report that FLT3-WT expressing cells do not express CXCR4 whereas the FLT3-ITD expressing cell line does (40). Nevertheless, an exogenous expression of FLT3-ITD in the HL-60 cells or inhibition of FLT3-ITD in the MV4-11 cells that might show a direct influence on CXCR4 were not conducted.

A recently published study by Jia et al. suggests that FLT3-ITD signaling could inhibit CXCR4 expression as it reports that treatment of FLT3-ITD expressing MOLM-14 cells with a FLT3 inhibitor resulted in an upregulation of CXCR4 on the cell surface, as well as on the mRNA level (35). If this mechanism proves true, the CXCR4 surface expression would increase during FLT3 inhibitor treatment, which might then lead to enhanced residing of the AML cells in the BMM. A combinatorial treatment with CXCR4 antagonists would therefore be mandatory to overcome the resistance to inhibitor treatment and avoid leukemia relapse.

## 5 Conclusions

It has been clearly shown that the level of CXCR4 expression in AML patient samples does have prognostic value and that targeting CXCR4 in AML could be a promising strategy to eliminate leukemic cells hiding in the bone marrow niche.

**Figure 1:**
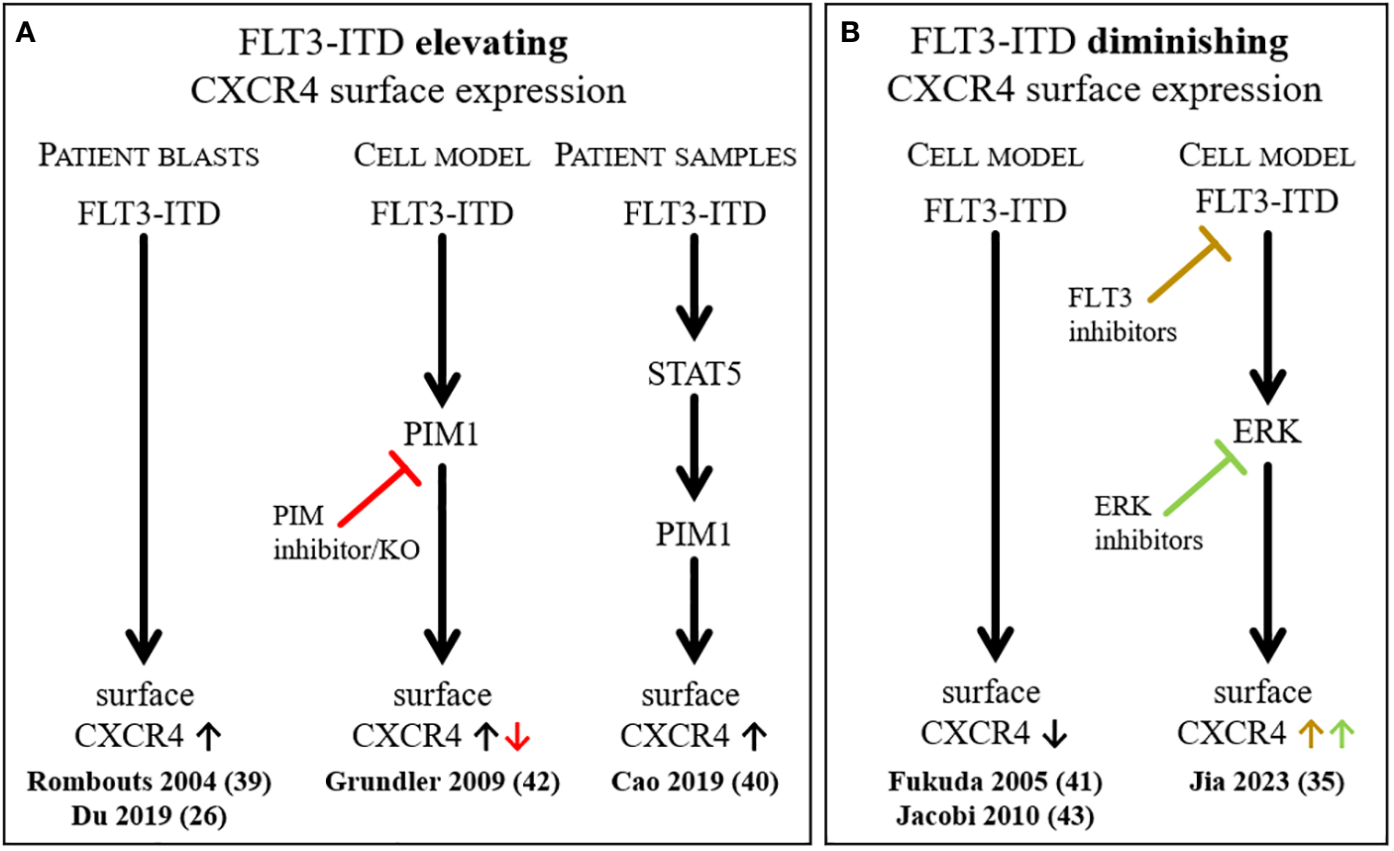
Summary of selected findings describing the influence of FLT3-ITD on the surface expression of CXCR4. Simplified depiction of literature reports on FLT3-ITD elevating **(A)** or diminishing **(B)** CXCR4 surface expression.

However, contrasting findings on the influence of FLT3-ITD on the surface expression of CXCR4 were reported and simplified results are summarized in **Figure 1**. The analysis of patient data indicates that the presence of FLT3-ITD leads to a higher CXCR4 expression, which was also supported by a cell model study (**Figure 1A**), but several reports of FLT3 overexpression experiments or FLT3 inhibitor usage point in the opposite direction (**Figure 1B**). All the above-mentioned studies show that the situation found in patients cannot easily be reproduced in cell culture experiments. Obviously, the complicated interplay of different stimuli and tissues in AML patients leads to the development of a specific cell phenotype that is perfectly adapted to evade the AML treatments.

In any way, the more detailed information would be available, the better the treatment strategies could be optimized to improve the prognosis of patients suffering from AML.

## Author contributions

JD: Conceptualization, Funding acquisition, Writing – original draft, Writing – review & editing. LK: Writing – original draft, Writing – review & editing.
